# Evaluation of Antibacterial, Antioxidant, Anti-inflammatory and Anticancer Efficacy of Titanium-Doped Graphene Oxide Nanoparticles

**DOI:** 10.7759/cureus.51737

**Published:** 2024-01-06

**Authors:** S Vishaka, S Nehal Safiya, M Binigha, Durai Singh Carmelin, P Geetha Sravanthy, Ramanathan Snega, Muthuvel Surya, Muthupandian Saravanan

**Affiliations:** 1 Department of Pharmacology, Saveetha Dental College and Hospitals, Saveetha Institute of Medical and Technical Sciences, Chennai, IND

**Keywords:** antioxidant, titanium/graphene nanoparticles, anti-inflammatory, antibacterial activity, anticancer

## Abstract

Introduction: The current development of nanoparticles (NPs) with significant antibacterial properties, low cost and low toxicity has made it possible to develop novel techniques for treatments in the medical field. The titanium metal oxide, when combined with a carbonaceous material like graphene, which has excellent absorbing capacity, is efficient in loading drugs and thus helps in drug delivery and also in biomedical applications like anticancer, anti-inflammatory, antioxidant, and antibacterial activities.

Materials and methods: Titanium-doped graphene oxide nanoparticles (Ti/GO-NPs) were processed by the one-pot synthesis method; further characterization was performed by using UV-visible spectroscopy, Fourier transform-infrared spectroscopy (FT-IR), field emission electron microscopy (FE-SEM), and energy-dispersive X-ray spectroscopy (EDX) analysis and biomedical applications like anticancer, anti-inflammatory, antioxidant and antibacterial activities.

Results: The synthesized end product of Ti/GO-NPs showed a creamy white appearance. Subsequent characterization studies of UV-Vis spectroscopy revealed a peak level of 373 nm at 24 hours and 404 nm after 48 hours. FT-IR analysis exhibited a broad absorption band within the range of 1000-3500 cm^-1^, which was attributed to various chemical compounds of C-Br, C-I stretching, C=C bending, S=O stretching, O=H stretching, C=C stretching, H bonded and OH stretching to different absorbance wavelength ranges. SEM analysis exhibited quasi-spherical-shaped Ti/GO-NPs with an average particle size of 50- 100 nm and EDX analysis showed the elemental composition of 32.3% titanium 43.9% oxygen and 2.5% carbon. The antibacterial activity showed moderate activity against *Staphylococcus aureus* and no activity against *Pseudomonas aeruginosa, Enterococcus faecalis and* *E. coli*. The antioxidant activity exhibited 88% at 50 µg/mL concentration, the anti-inflammatory activity revealed 80% at 80 µg/mL concentration and the anticancer activity showed 21% at 150 µg/mL concentration.

Conclusion: The characterization and biomedical application conclude that a combination of Ti/GO-NPs will be efficient in drug delivery. The study showed moderate antibacterial activity and significant antioxidant, anti-inflammatory and anticancer activities. Considering their physiochemical properties, absorption capacity and mechanism of drug delivery, Ti/GO-NPs can be incorporated into various applications in the medical field.

## Introduction

Nanoparticles (NPs) comprise particular elements with at least a single dimension below 100 nm [1]. Nanomaterials are widely used in many fields for both diagnostic and therapeutic medical applications. Depending on their size and surface functions, NPs display a wide range of diverse characteristics [2]. Therapeutic NPs aim to increase the concentration and dispersion of substances with medicinal properties at the site of infection, improve treatment efficacy and reduce the frequency and severity of side effects [3]. Titanium NPs offer a wide range of applications and hold promise for advancements in medicine, electronics, energy and environmental science. Titanium is frequently used in dental implants due to its advantageous chemical and mechanical properties. Different kinds of titanium nanostructured material delivery systems have been created for the effective killing of tumor cells by chemotherapy drugs [4]. Strong reactivity of titanium (Ti) with oxygen when exposed to air leads to the production of a chemically stable reducing oxide layer, which contributes to Ti's biocompatibility. When compared to other metal alloys, Ti is more biocompatible, and thus, Ti is often utilized in biocompatible metallic biomaterials [5]. Graphene, which is well known for its structural properties, helps in the carrying of drugs in the form of nanocomposites, especially the structure of sp^2^ hybridization, hexagonal arrangement, and crystal lattice in the form of a honeycomb structure [6]. Graphene is available in different base materials, namely, graphene oxide (GO), reduced graphene oxide (rGO) and graphene quantum dots. It has the capacity to make the doped NPs an effective drug carrier because of their large surface area [7]. It has a broad range of oxygen-containing functional groups such as carboxyl, hydroxyl and keto groups on its surface. Bioapplications comprise drug delivery of minute molecules like drugs or genes, biosensing, tissue engineering, bioimaging, and photothermal therapies [8]. Physical methods, including mechanical milling, sonication, physical vapor deposition and laser ablation, were costly and required sophisticated electrical equipment [9]. Ball milling, sol-gel, spray drying and solvothermal and hydrothermal processes are some of the methods used in chemical synthesis [10]. In order to shift to an eco-friendly method, one-pot synthesis will be the best solution, which is also cost-effective. In comparison to the other synthesis studies, previous research articles have shown that one-pot synthesis will be a cost-effective approach for the synthesis of NPs and will also help in large-scale production [11,12]. Reactive oxygen species (ROS) are able to pass only through the layer of peptidoglycan, and thus the process of antibacterial activity occurs in the outer and inner membranes [13]. ROS also play a major in oxidative stress, which is responsible for the inflammation of the infected site [14]. There is a strong connection between graphene biocompatibility and an increase in solubility; therefore, it is essential to modify the surface characteristics of GO and its cytotoxic action by modifying GO sheets using different biomaterials [15]. One-pot synthesis has become an effective synthesis method as it does not require intermediate compound production or purification. Thus, by this one-pot synthesis method, Ti/GO-NPs will be synthesized and subjected to characterization by using UV-vis, FT-IR, FE-SEM and EDX and biomedical applications such as antibacterial, antioxidant, anti-inflammatory and anticancer efficacy.

## Materials and methods

Materials and chemicals

Titanium(IV)dioxide (TiO_2_), graphene oxide (GO), bovine serum albumin (BSA), diclofenac sodium, Muller-Hinton agar (MHA), sodium hydroxide (NaOH), 0.5 McFarland, resazurin dye, and dimethyl sulfoxide (DMSO) were purchased from Hi-Media, India. Bacterial strains of *Staphylococcus aureus, Enterococcus faecalis, Pseudomonas aeruginosa* and *E. coli *were obtained from the Medical Microbiology Lab of Saveetha Medical College, Chennai. A lung cancer cell (A-549) was obtained from the NCCS (National Centre for Cell Sciences), Pune, India.

Synthesis of titanium-doped graphene nanoparticles

Processing of Ti/GO-NPs was done by the one-pot synthesis method; stock solution preparation was carried out with 0.1M GO, 0.1M TiO_2_ and 0.1M NaOH. 0.1M 50 mL of graphene was taken in a burette along with 0.1M 5 mL of NaOH, due to their alkaline nature which not only acts in the speeding up of the reduction process but also the enhancement of oxidation take place, one possible reaction that occurs is the reduction of graphene oxide (GO) by NaOH. This reduction process converts GO into reduced graphene oxide (rGO), which serves as a support material for the TiO_2_ NPs [16]. Another burette was filled with 50 mL of TiO_2_. Furthermore, the conical flask with the graphene solution was placed in a magnetic stirrer. The magnetic stirrer was kept at 1000 rpm, both burettes were opened simultaneously, and the content was added dropwise. The reaction begins and the change in color appears slowly from black to creamy white within 12 minutes, which indicates the formation of Ti/GO-NPs. The prepared solution was placed in a shaker to complete the reaction. Ti/GONPs were centrifuged at 6000 rpm for 15 minutes at 4°C. Before filtration, the mixture was rinsed with distilled water in order to remove any adhering contaminants. The moisture content was then completely extracted by heating the mixture overnight in a hot air oven at 70 °C. The Ti/GO-NPs were collected and stored in vials after the moisture evaporated. The purity level of the synthesized nanoparticle was analyzed by EDX.

Characterization of Ti/GO-NPs

After 24 hours of synthesizing Ti/GO-NPs, the characterization of the Ti/GO-NPs was done by spectroscopic and microscopic techniques. Particle size, elemental mapping and surface morphology were recorded using UV-Vis spectroscopy (Labman Double Beam UV-vis spectrophotometer LMSPUV1900S, India, 190-1100 nm) with absorbances of the wavelength range from 190 to 1100 nm, FT-IR (Bruker Alpha II, Germany) with absorbances of the wavelength range of 1000-3500 cm^-1^, FE-SEM (JEOL-800S), and EDX (OXFORD X-Plore-30/C-Swift, Oxford Instruments, Oxfordshire) to evaluate the chemical element composition of the NPs present.

Antibacterial efficacy studies (agar well diffusion method)

The agar well diffusion technique was performed to observe the zone of inhibition by Ti/GO-NPs against bacterial strains, namely, *Escherichia coli*,* Pseudomonas aeruginosa, Staphylococcus aureus *and *Enterococcus faecalis*. The bacterial strains were grown in MHB for 18 hours at 37°C and adjusted to the 0.5 McFarland standard. A sterile MHA plate was prepared by dissolving MHA in 300 mL of distilled water and kept in an autoclave for 20 minutes at 121 °C for sterilization. Later, the prepared plate was swabbed with the respective bacterial strains. The 2 of 11 diffusion wells were punched using the sterile tips and added with Ti/GO-NPs at different concentrations (10, 20, 30, 40 and 50 µg/mL) which represent (m1, m2, m3, m4, m5) and control (Streptomycin), respectively. This study was carried out in a dose-dependent antibacterial activity in terms of the zone of inhibition since the Ti/GO-NPs were taken in different concentrations (10, 20, 30, 40 and 50 µg/ml) compared with the control Streptomycin (30 µg/ml). Streptomycin exhibits a wide-ranging antibacterial activity, proving effective against both gram-positive and gram-negative bacteria, as demonstrated in comparison to other research literature. Given its broad-spectrum nature, the test incorporated a positive control [17]. The plates were incubated for 24 hours, and the zone of inhibition was observed.

Antioxidant activity

The antioxidant activity of Ti/GO-NPs was analyzed spectrophotometrically by the DPPH (2,2-diphenyl-1-picrylhydrazyl) radical scavenging assay. The antioxidant activity is typically dose-dependent; as the concentration of the NPs increases, the antioxidant activity also increases. The ability of antioxidants to supply hydrogen is assumed to be responsible for their influence on DPPH radical scavenging. DPPH is a stable free radical that receives an electron or a hydrogen radical to form a diamagnetic molecule [18]. A 96-well microtiter plate was used for this procedure. The well was loaded with Ti/GO-NPs (20, 40, 60, 80 and 100 µg/mL) and then 95% ethanol was added to 80, 60, 40 and 20 µl respectively to make up the volume for 100 µl. Furthermore, each well was added with DPPH and 100 µl of ethanol and 100 µl of DPPH were used to maintain as a control. The standard was prepared by adding ascorbic acid, the same as the concentration of Ti/GO-NPs (20, 40, 60, 80 and 100 µg/mL) and DPPH was added to make up the volume of 100 µl. Incubate the plate in a dark room for 30 minutes. The absorbance of the peak wavelength of 517nm using an (enzyme-linked immunosorbent assay) ELISA reader.

Anti-inflammatory activity 

The anti-inflammatory activity procedure was carried out by an albumin denaturation assay on a microtiter plate with minor modifications. The biological activity involved in the anti-inflammation is due to the cyclooxygenase enzyme which has the active site for the binding of the anti-inflammatory drug [19]. The pH was adjusted to 6.8 using 1N hydrochloric acid (HCl) and 1% BSA (90, 80, 70, 60 and 50 µl) along with different concentrations of Ti/GO-NPs (10, 20, 30, 40 and 50 µg/mL) to make the concentration as 100μg/mL. As a negative control, 50 µl of DMSO and 50 µl of BSA were taken; diclofenac sodium is used as the standard positive control. This experiment was triplicated. The reaction solution was incubated for 15 mins at ambient temperature and again incubated for 20 minutes at 55°C. The absorbance of wavelength ranges was noted at 660 nm and the inhibitory percentage was calculated.

Anticancer activity

The anticancer activity of Ti/GO-NPs for lung cancer cells was performed by a 3-(4,5-dimethylthiazol-2-yl)-2,5-diphenyl tetrazolium bromide (MTT) assay. The anticancer activity responds to the lung cancer cells dose-dependent with cell viability, as the concentration of the nanoparticles increases the cancer cell viability decreases. The biological activity is carried out by the enzymatic reduction when viable cells are exposed to MTT, the molecule undergoes reduction to form formazan [20]. The T-25 flask, which contains DMEM with dispersed cells, is populated with 10% FBS. As the cells reached a confluency rate of 70%, the cells were seeded in 96-well microtiter plates and treated with Ti/GO-NPs in various concentrations of (10, 20, 40, 60, 80 and 100 µg/mL) and incubated at room temperature for 24 hours. After the period of incubation, the mean and standard deviation (SD) were calculated based on the standard operating procedure.

Statistical analysis

The experimental data underwent statistical analysis using one-way ANOVA with a significance level of α=0.05. Microsoft Excel 2016 (Microsoft Corporation, Redmond, USA) was utilized for this analysis. Each experiment was independently conducted three times and subsequently, Dunnett's test was employed to assess the significance of the difference between the standard and treatment groups. A significance level of p≤0.05 was considered statistically significant.

## Results

Synthesis of Ti/GO-NPs

**Figure 1:**
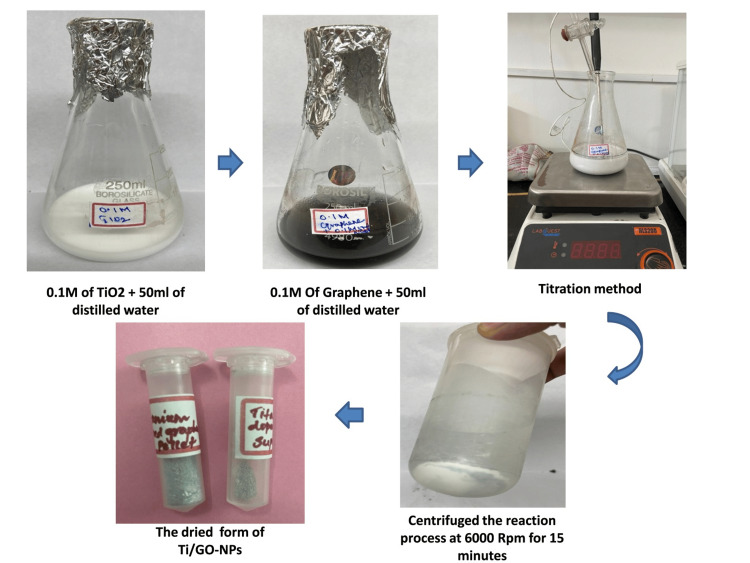
Overview of synthesized titanium-doped graphene oxide nanoparticles that showed black to creamy white color change

The Ti/GO-NPs were produced by the one-pot synthesis method and the color change was observed from black to creamy white after 48 hours of reactions and creamy white color change indicates the synthesis of TiO/GO NPs (Figure 1).

UV-visible spectroscopy

**Figure 2:**
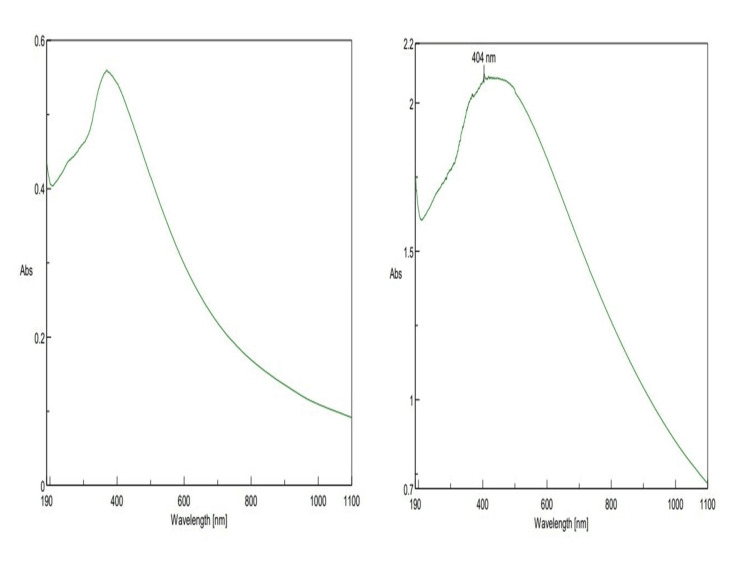
Ultraviolet visible spectroscopy of titanium-doped graphene oxide nanoparticle absorbance 373 nm at 24 hours (T24) and 404 nm at 48 hours (T48)

Synthesized Ti/GO-NPs were analyzed using UV-Vis spectroscopy. The spectrophotometer measures plasma resonance band absorbance of wavelengths ranging from 190 to 1100 nm, at 24 hours, the peak absorption of wavelength was found to be 373 nm, and after 48 hours, the peak was at 404 nm (Figure 2). With this reading, the presence of Ti/GO-NPs was confirmed.

Fourier transform infrared spectroscopy (FT-IR)

**Figure 3:**
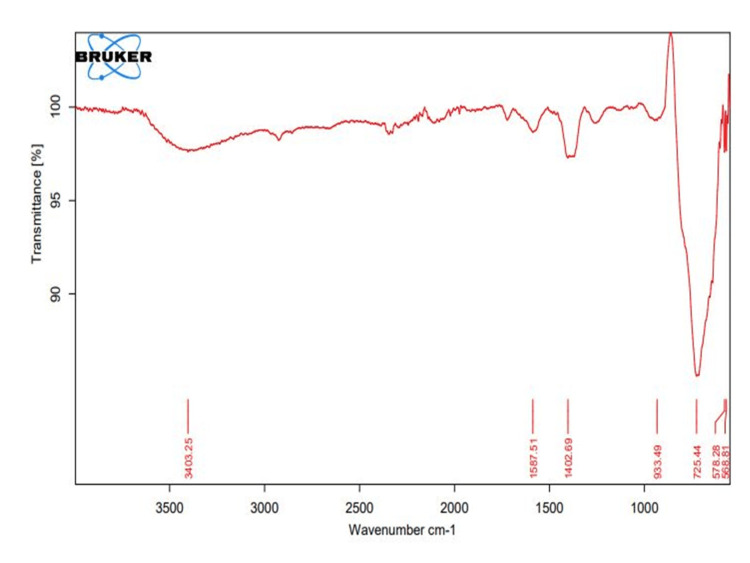
Fourier transform infrared spectroscopy absorbance of elemental composition of titanium-doped graphene oxide nanoparticles in the wavelength range within 1000-3500 cm-1

By using the FT-IR analysis, a broad absorption band was found within the range of 1000-3500 cm^-1^, which was attributed to the halo compound stretching mode of C-Br, C-I stretching found at a wavelength of 568.81 cm^-1^ and 578.28cm^-1^, and C=C bending of alkene compound distributed at a wavelength of 725.44 cm^-1^, The absorbance in the wavelength range of 1402.69 cm^-1^ showed the S=O stretching of the sulfate compound and O=H stretching of alcohol compounds 1587.57 cm^-1^ wavelength peak absorbance was at the C=C stretching of α,β unsaturated ketone compounds. Alcohol and hydroxy compounds of the hydroxy group, H-bonded OH stretch, are found at an absorbance wavelength of 3403.25 cm^-1^ (Figure 3).

Field-emission scanning electron microscopy (FE-SEM)

**Figure 4:**
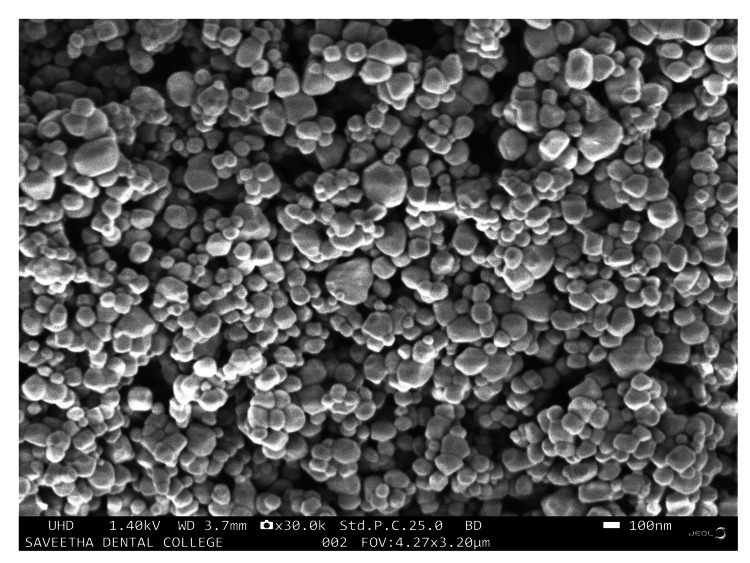
Field-emission scanning electron microscopy analysis showed agglomerated quasi-spherical shaped titanium-doped graphene oxide nanoparticles

The FE-SEM analysis was done at three different magnifications 0.5 μm, 1 μm and 100 nm. The structural characteristics of Ti/GO NPs were found to be agglomerated, quasi-spherical shaped and at 100nm magnification. The average particle size of Ti/GO NPs ranged between 50 and 100 nm (Figure 4).

Energy-dispersive X-ray spectroscopy (EDX)

**Figure 5:**
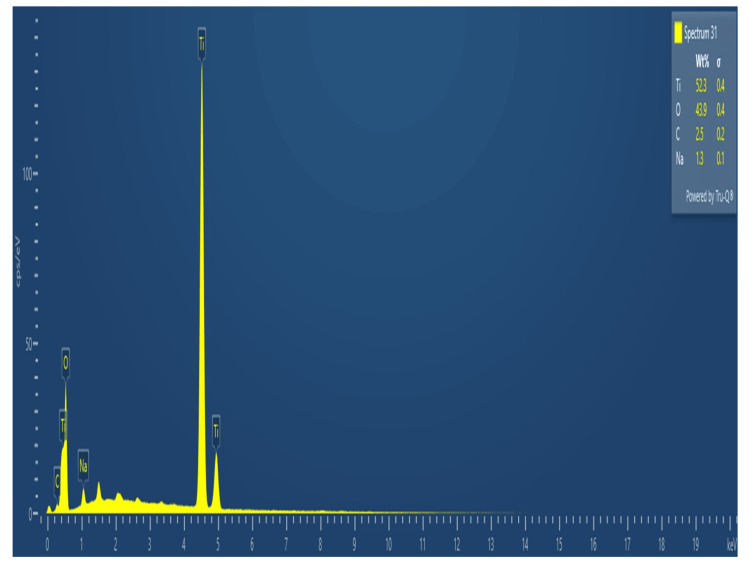
Energy-dispersive X-ray spectroscopy elemental composition analysis of titanium-doped graphene oxide nanoparticles

The elemental compositions of the produced Ti/GO-NPs were verified by using EDX and Figure 5 shows the peaks that were acquired. It has been noted that the prepared Ti/GO-NPs are mostly made of Ti, O and C, with no evidence of additional elements. The presence of carbon indicates the presence of graphene. The percentage of elemental composition present in the Ti/GO-NPs namely, Ti, O, and C is at 32.3%, 43.9%, and 2.5%, respectively.

Antibacterial activity

**Figure 6:**
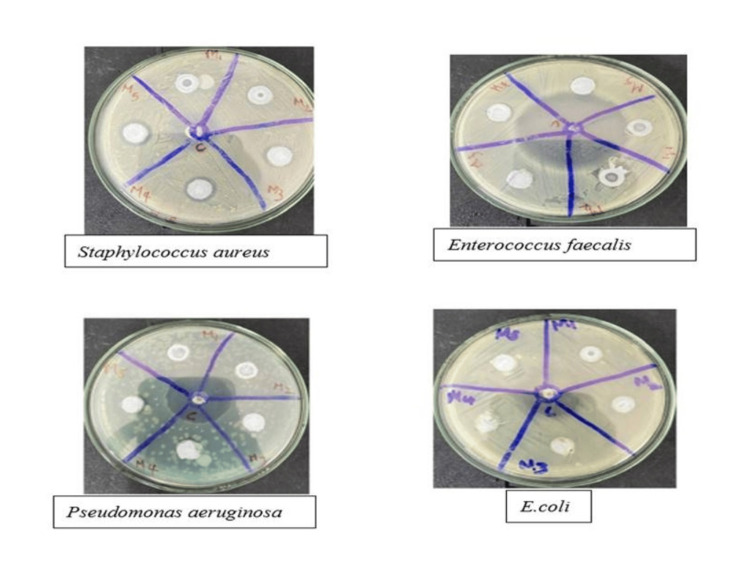
Antibacterial activity against the selected bacterial pathogens

The antibacterial activity was measured by the agar-well diffusion method with Ti/GO-NPs, and the zone of inhibition was measured. Moderate activity was seen against *Staphylococcus aureus,* and no zone of inhibition was found against *Enterococcus faecalis,* *Pseudomonas aeruginosa* and *E. coli *(Figure 6).

Antioxidant activity

**Figure 7:**
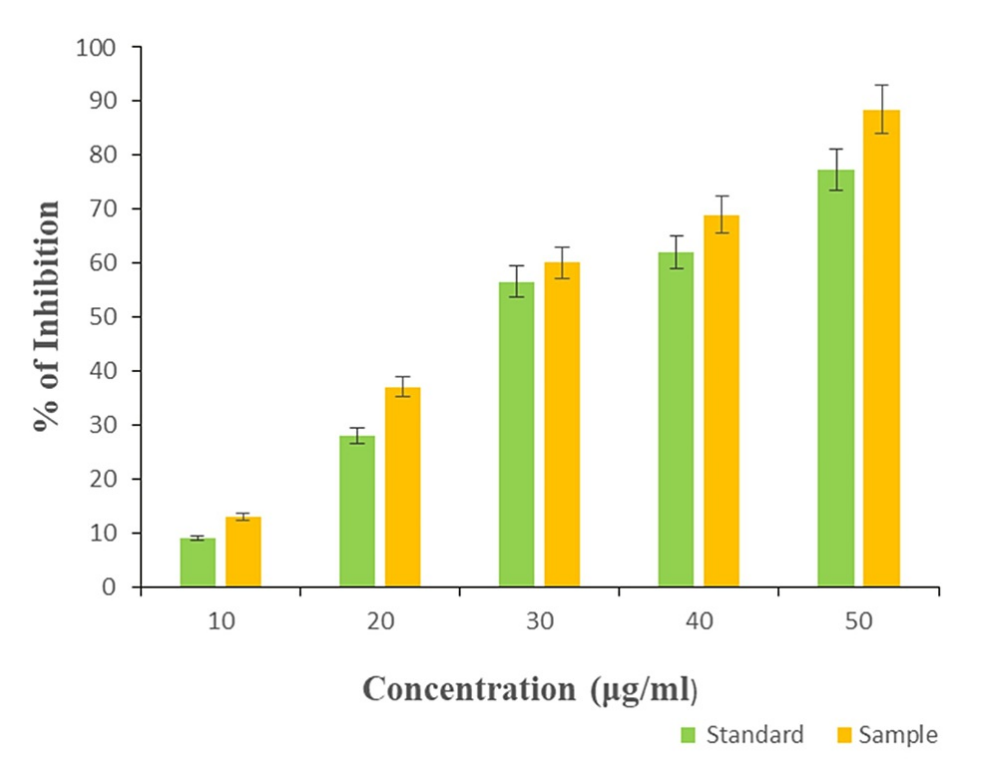
: Graphical representation of antioxidant activity of titanium-doped graphene oxide nanoparticles. Data are presented as means ± standard deviations (n=3) compared to the standard group, p≤0.05 was considered statistically significant

The antioxidant activity was assessed using the DPPH (2,2-diphenyl-1-picrylhydrazyl) assay, and the experiment was conducted in triplicate. The mean value was calculated, and a significance level of p≤0.05 was used to determine statistical significance. These results were then presented graphically. The graphical representation of synthesized Ti/GO-NPs showed 88% of maximal antioxidant activity at 50µg/ml of concentration when compared with the standard ascorbic acid (75% activity), 60% of moderate activity at 30µg/ml, and 12% of minimal activity at 10µg/ml (Figure 7).

Anti-inflammatory activity

**Figure 8:**
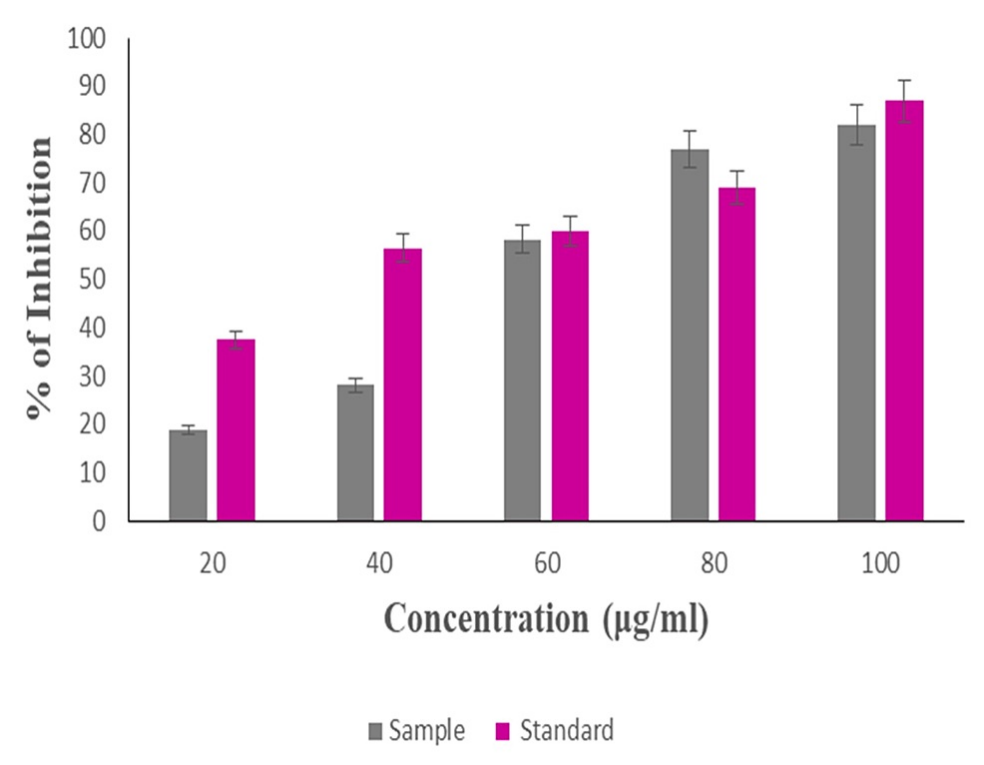
Graphical representation of anti-inflammatory activity of titanium-doped graphene oxide nanoparticles. Data are presented as means ± standard deviations (n=3) compared to the standard group; p≤0.05 was considered statistically significant.

The anti-inflammatory activity was evaluated through the albumin denaturation assay, and the experiment was triplicated. The mean value was computed, and the significance level of p≤0.05 was employed to determine the statistical significance. These findings were subsequently depicted in a graphical format. As illustrated in this graphical representation, the synthesized Ti/GO-NPs had a noteworthy anti-inflammatory activity of 80% at a concentration of 80µg/ml. Additionally, the 20µg/ml concentration of Ti/GO-NPs exhibited a minimum activity of 19% when compared to the standard drug Diclofenac Sodium, which demonstrated 70% and 38%, respectively (Figure 8).

Anticancer activity

**Figure 9:**
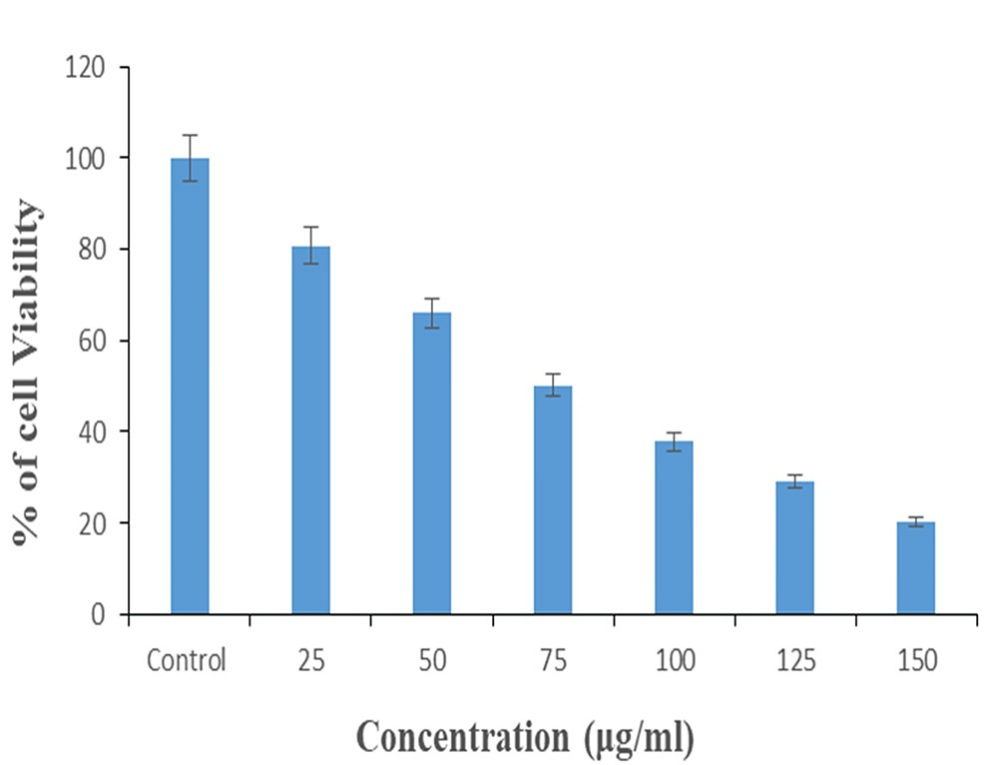
Graphical representation of 3-(4,5-dimethylthiazol-2-yl)-2,5-diphenyl tetrazolium bromide (MTT) assay of the lung cancer cell line at different concentrations of titanium-doped graphene nanoparticles. Data were represented in mean ± S.D (n=3), compared to the control group, p≤0.05 was considered statistically significant

The 3-(4,5-dimethylthiazol-2-yl)-2,5-diphenyl tetrazolium bromide (MTT) assay was performed using Ti/GO-NPs, which were examined against lung cancer cells and the experiments were carried out in triplicate and the significant mean value was calculated as p≤0.05. The anticancer activity is dose-dependent as the concentration of the NPs increases, the cell viability decreases to 21% at 150 µg/mL as shown in the graphical representation in Figure 9.

## Discussion

The synthesis of Ti/GO NPs was carried out by the one-pot synthesis method. Other studies explained that the synthesis of metal oxide NPs can be done by hydrothermal procedures [21]. When compared with this study, the one-pot synthesis method yielded an eco-friendly nanoparticle that is cost-effective, whereas the hydrothermal method requires expensive instruments, high temperatures, and chemical involvement.

The synthesis of Ti/GO-NPs resulted in a color change from black to creamy white, whereas the other study of synthesized TiO_2_ NPs with the plant extract *Moringa oleifera* showed a color change from white to pink-brown because of their reducing and stabilizing potential along with the involvement of secondary metabolites; however, the common change in color is white, which indicates the titanium NPs.

This may be due to the involvement of plant extract the combination of the plant extract phytochemicals and the nanoparticles reducing properties that lead to the color change. Based on the color change, further confirmation is done by the absorbance of the specific color range. In observation of size and shape exhibited by Ti/GO nanoparticles exhibited a quasi-spherical shape, whereas the synthesis of TiO_2_ NPs along with *Moringa oleifera* plant extract displayed irregular and anisotropic properties [22].

In our study, the Ti/GO exhibited 373nm at initial and, after 24 hours, 404nm due to the bio-reduction of the synthesized nanoparticles. When compared to *Echinacea purpurea*, it showed 280 nm, as the UV-Vis spectroscopy exhibited absorbance peaks only for the titanium nanoparticles [23]. From this study of comparison, it is clear that the maximum absorbance of nanoparticles depends on the particle size and band gap energy.

FT-IR analysis showed the absorbance wavelength range at 725.44 cm^-1^ C=C bending of the alkene compound distributed. Other similar studies showed the C=C stretching vibration presence of an absorption band around 1630 cm^-1^ of TiO graphene oxide [24]. In comparison, this study has the same chemical bonding of C=C stretching at the peak of wavelength, which is confirmed by the elemental chemical bonding of Ti/GO-NPs.

In comparison with this study, FE-SEM revealed an agglomerated, quasi-spherical shape with an average particle size of 50-100 nm. A similar study of the SEM image showed the morphology of the obtained TiO_2_-AgNS photocatalyst; TiO_2_ NPs are visible as white objects with a quasi-spherical shape. The average particle size estimated for Ag NSs was 18 ± 3 nm [25]. The shape of the titanium can be influenced by the graphene oxides, this is maybe due to the structure of graphene that differs from the titanium since graphene structure is said to be a two-dimensional plate-like shape, whereas the titanium NPs exhibit sphere shape when both the metals where brought into the combination it may exert in a quasi-spherical shape structure [26]. This comparison of both studies showed that the combined effect of graphene and TiO_2_ nanoparticles provides a quasi-spherical shape structure with a suitable surface roughness, which allows for optimal air-layer entrapment [27].

The EDX study of Ti/GO-NPs showed that the elemental composition of Ti, O and C was found to be 32.3%, 43.9% and 2.5%. When compared with the study of zinc-doped micro-arc titanium oxide, the elemental composition of Ti, O and C was 58.37%, 11.17% and 3.46% [28]. By this study, it can be confirmed that the required elemental composition is present and it is also evident that there are no other impurities, the purity level of the synthesized nanoparticle was analyzed by EDX.

Certain studies stated that the surface electric charge of the bacterial membrane can generate a change in its penetrability, ultimately leading to bacterial death. Our antibacterial study showed moderate activity against *Staphylococcus aureus* compared to the other organisms. Similar studies of the antibacterial activity of 9 of 11 silver-doped titanium oxides (Ag@TiO_2_) showed an inhibition zone of 22.3mm for *Staphylococcus aureus *[29]. The surface charge of the titanium is said to be strongly positively charged [30] and the graphene oxide surface charge is generally found to be negative, but due to the amidation reaction, they tend to change as positively charged. The interaction between the positively charged nanoparticles and the negatively charged bacterial cell wall leads to the adhesion, penetration and destruction of the bacterial cells [31]. Another factor of activity is because it may depend on the concentration-dependent anti-microbial activity against* Staphylococcus aureus* when compared with Streptomycin. For example, one of the main components of the cell wall of *Staphylococcus aureus *is teichoic and lipoteichoic acid, which results in a negatively charged surface-resistant nature [32].

Ti/GO-NPs have the tendency to enhance hydrophobicity as well as strong corrosion resistance and low toxicity. Graphene and titanium dioxide are introduced as coating materials. By suppressing the productivity of reactive oxygen species of bacterial pathogens through photocatalytic activity, antibacterial activities can be performed [25]. The combination of the titanium and graphene oxide provides a synergic effect by reducing the surface wettability by increasing the surface roughness based on the water contact angle by increasing the concentration of the nanoparticles. This hydrophobic coating and strong corrosion resistance of the implants and medical equipment results in biofilm-free devices in the future [33]. As a result, enhancing the coated surface with superhydrophobic properties may show good application in orthopedic implants and drug delivery systems.

Ti/GO-NPs showed 88% for 50 µg/mL, thus Ti/GO-NPs resulted in good antioxidant properties when compared to the antioxidant studies carried out with graphene doped for various metal oxides when compared with the standard ascorbic acid which showed 10-50% at 15-65 µg/mL [34]. The antioxidant activity is typically dose-dependent in our study; as the concentration of the nanoparticles increases, the antioxidant activity also increases. As shown in the graphical representation, the synthesized Ti/GO nanoparticles show greater antioxidant activity than the standard ascorbic acid.

Inflammatory progress occurs through various types of pathways; the important inflammatory pathway involves nuclear factor kappa-light-chain-enhancement of activated B cells. It is necessary to provide stimuli to respond to inflammation, stress and oxidized low-density lipoprotein (LDL), or bacterial antigens. Our study showed 80% of maximum anti-inflammatory activity at 80 µg/mL compared to the standard, when compared to the other study of TiO_2_-ZnO NC exhibited 83% of activity at 80 µg/mL concentration [35] which is similar to our study. This helps to understand the activity of titanium nanoparticles.

Thus, Ti/GO-NPs showed efficient activity at lower concentrations. Our study of anticancer activity showed 21% for Ti/GO-NPs at 150 µg/mL. Other similar studies showed that lung cancer cells treated with GO showed anticancer activity of 21.69% at 1000 µg/mL [36]. The anticancer activity responds to the lung cancer cells in a dose-dependent manner with cell viability, as the concentration of the nanoparticles increases the cancer cell viability decreases. Compared with the evaluation of the cytotoxicity and oxidative stress response of CeO-RGO nanocomposites in human lung epithelial A549 cells, our study of Ti/GO NPs showed significant anticancer activity when compared to Ce-O_2_ RGO, whereas Ce-O_2 _RGO showed no significant change in toxicity. The graphene oxide was found to induce dose-dependent cytotoxicity. This study showed that graphene and TiO_2_-based nanostructures are therapeutic materials in cancer treatment [37]. With this comparison, it can be understood that the incorporation of graphene with titanium increases the anticancer therapeutic activity. According to this comparison, Ti/GO-NPs showed significant anticancer activity at this lowest concentration (150 µg/mL).

Limitations

In the current study, we reported several types of in vitro analyses to evaluate the titanium-doped graphene oxide NPs synthesized from the plant extract. Additional in vivo research, such as animal and clinical trials, will be helpful in better understanding its effects.

## Conclusions

The study provides a brief overview of Ti/GO-NP's various properties; synthesis of Ti/GO-NPs was performed by an eco-friendly one-pot synthesis method and the characteristics Ti/GO-NPs are identified as quasi-spherical-shaped and components of various chemical bonding confirm the presence of Ti/GO-NPs. They also exert moderate antibacterial activity against *Staphylococcus aureus* and no activity against *Enterococcus faecalis, Pseudomonas aeruginosa* and *E. coli*. Furthermore, they revealed significant antioxidant, anti-inflammatory and anticancer activity. This study concludes that combining titanium metal oxides and graphene with strong structural properties will provide a better solution for nanotherapeutics and drug delivery systems.
